# The Gastrointestinal Tumor Microenvironment: An Updated Biological and Clinical Perspective

**DOI:** 10.1155/2019/6240505

**Published:** 2019-11-22

**Authors:** Silvia Batista, Ana C. Gregório, Andreia Hanada Otake, Nuno Couto, Bruno Costa-Silva

**Affiliations:** ^1^Champalimaud Research, Champalimaud Centre for the Unknown, 1400-038 Lisbon, Portugal; ^2^Center for Translational Research in Oncology (LIM-24), Instituto do Câncer do Estado de São Paulo, Hospital das Clínicas (HCFMUSP) Faculdade de Medicina da Universidade de São Paulo, 01246-000 São Paulo, Brazil; ^3^Digestive Unit, Champalimaud Clinical Centre, 1400-038 Lisbon, Portugal

## Abstract

Gastrointestinal cancers are still responsible for high numbers of cancer-related deaths despite advances in therapy. Tumor-associated cells play a key role in tumor biology, by supporting or halting tumor development through the production of extracellular matrix, growth factors, cytokines, and extracellular vesicles. Here, we review the roles of these tumor-associated cells in the initiation, angiogenesis, immune modulation, and resistance to therapy of gastrointestinal cancers. We also discuss novel diagnostic and therapeutic strategies directed at tumor-associated cells and their potential benefits for the survival of these patients.

## 1. Introduction

Gastrointestinal (GI) cancers represent the most prevalent tumors worldwide and the major cause of death related to cancer. Within this group, we can identify colon, stomach, and liver cancers as the main concerns according to their prevalence (fourth, sixth, and seventh most prevalent, respectively). As a cause of death, stomach (second higher), liver (third higher), and colon cancer (fifth higher) are the main culprits [1]. Nonetheless, this group includes other mention-worthy cancers. Although not within the ten most prevalent, pancreatic ductal adenocarcinoma (PDAC) has one of the worst prognoses and is expected to be one of the major causes of death related to cancer by 2030 [2]. Additionally, the esophagus cancer is highly prevalent in some areas of the globe [1].

The treatment outcomes are also completely different amongst GI cancers. Two of the main reasons concern the timing of diagnosis and the therapeutic approach. As an example, colon and rectum cancers are usually diagnosed at early stages and are treated with surgery (colon cancer [3]), or multimodality treatment including chemoradiotherapy and surgery (rectum cancer [4]) with high rates of success, especially in the latter. Nonetheless, other tumors such as PDAC are usually diagnosed at later stages, when surgery approaches are usually no longer feasible. In these cases, the traditional treatment is based on combined chemotherapy [3–7], with 5 years below 5% [8].

The development of a prominent desmoplastic reaction by both local and distantly recruited stromal cells has been observed in GI cancers. In addition to immune cells, bone marrow- (BM-) derived progenitor cells are recruited to the tumor microenvironment (TMEN) where they differentiate into various stromal cells, such as endothelial cells, pericytes, and fibroblasts [9]. These cells are crucial for both malignization and cancer progression [10] and are frequently associated with poor prognosis [11–14]. Indeed, the interaction of cancer cells and the host microenvironment plays a critical role in strengthening the metastatic proficiency. Thus, a better understanding of the oncological drivers of these tumors, including their interaction with the microenvironment, is of utmost importance [15, 16]. In this review, we will focus on the role of these tumor-associated cells in the tumorigenesis and progression of GI cancers, as well as on their role in treatment resistance and potential targeted therapeutic approaches.

## 2. BM-Derived Progenitor Cells

BM-derived cells (BMDCs) are constantly recruited to the TMEN, where they modulate tumor growth and metastasis through the regulation of angiogenesis, inflammation, and immune suppression [17]. Several studies in animal models have implicated BMDCs in the development of carcinomas of the upper GI tract [18, 19], including gastric cancer (GC) [20]. BMDCs were shown to home and repopulate the gastric mucosa in response to *H. pylori* chronic infection, leading to the development of metaplasia, dysplasia, and cancer over time [20]. In another study, BMDCs were found to be about 25% of *H. pylori*-induced dysplastic lesions in a mouse model [21]. Zhang et al. showed that highly metastatic colorectal carcinoma (CRC) cells produce elevated serum levels of OPN, MMP9, S100A8, S100A9, SAA3, and VEGFA. This promoted the setup of hepatic TMENs supportive of metastasis by BMDCs recruitment to this organ [22]. Bone marrow-derived CD34^+^ CD31^−^ immature myeloid cells were also found to cluster at the invasion front of CRC in cis-Apc^+^/D716 Smad4^±^ mutant mice. These immature myeloid cells expressed Metalloproteinases (MMP) 9 and MMP2 and supported tumor invasion at early stages in intestinal adenocarcinomas [23].

Bone marrow-derived mesenchymal stem cells (MSCs) constitute a nonhematopoietic stem cell subpopulation that can populate the TMEN and contribute to tumor growth and progression through paracrine signaling [24]. Data from Beckermann et al. suggested a supportive role of MSCs in angiogenesis [25]. In this study, MSCs display increased vascular endothelial growth factor (VEGF) mRNA expression and protein secretion. They were also found to migrate towards tumor blood vessels of PDAC, *in vitro* and *in vivo*, in response to tumor-secreted growth factors. This is reinforced *in vivo* in an orthotopic mouse model of PDAC, where siRNA directed towards VEGF induces loss of vessel density control by MSCs [25].

Hypoxia can also induce the expression of growth factors that act as chemoattractants for MSCs to the TMEN [26–29]. In fact, the expression of VEGF by MSCs was shown to increase upon stimulation by interferon- (IFN-) *γ* and tumor necrosis factor- (TNF-) *α* cytokines through a hypoxia-induced factor- (HIF-) 1*α*-dependent pathway. This promoted angiogenesis and tumor growth in mice bearing CRC [30].

Some reports also implicate MSCs in tumor progression and metastasis. For instance, S100 Calcium Binding Protein A4 (S100A4) secreted by MSCs isolated from patient-derived hepatocellular carcinoma (HCC) tissues upregulated the expression of miR155 in HCC cells, promoting tumor invasion through the suppressor of cytokine signaling 1- (SOCS1-) MMP9 axis [31]. Exosomes, extracellular vesicles of endosomal origin, can mediate transfer of biomolecules both locally and at distance, playing a key role in the setup of TMENs [32, 33]. For instance, MSCs-derived exosomes supported GC lymph node metastasis and venous invasion by transferring miR-214, miR-221, and miR-222, regulators of the tumor suppressor gene Phosphatase and Tensin Homolog (*PTEN*), to cancer cells [34].

Bone marrow-derived MSCs also have the ability to differentiate into several cell types in the stroma [24], including fibroblasts [35]. Spaeth et al. showed the propensity of MSCs to transition to a tumor-associated fibroblast-like phenotype in ovarian, breast, and PDAC-xenografted tumors. These fibroblast-like cells ultimately contributed to microvascularization and the production of tumor-stimulating paracrine factors [36]. MSCs also favor primary CRC cells metastasis to the liver [37]. Orthotopic transplantation of cancer cells mixed with MSCs (but not cancer cells on their own) into the cecal wall resulted in macroscopic liver metastasis. Interestingly, metastasized colon cancer cells recruited more MSCs to the secondary sites where these were found to differentiate into supporting fibroblast‐like cells [37]. Altogether, these results illustrate the role of MSCs in the development of tumor-supporting microenvironments.

Hematopoietic stem cells (HSC) constitute a subpopulation of BM resident cells endowed with long-lived self-renewal and multipotency that sustain the generation of all cells of the blood and immune system. The HSC niche is tightly regulated by osteoclasts and vascular cells within the BM compartment, contributing for the maintenance of a quiescent microenvironment and controlled differentiation [38, 39]. However, tumor-derived soluble factors are able to systemically induce a BM microenvironment switch, from quiescent to protumorigenic and proangiogenic, and stimulate HSC mobilization into the circulation and recruitment to the tumor [39]. These cells can indirectly modulate tumor growth through their ability to differentiate into myofibroblasts and inflammatory cells in the tumor environment.

## 3. Cancer-Associated Fibroblasts (CAFs)

Fibroblasts are normal components of the connective tissue. These spindle-shaped cells are the main nonepithelial and nonimmune cell elements found in the interstitial space, embedded in physiological extracellular matrix (ECM) [40]. Resident fibroblasts of healthy tissues are considered to be in a resting or quiescent state and are characterized by low metabolic and synthetic activities. In the physiological wound healing process, resting fibroblasts become activated, gaining contractile properties and becoming synthetically dynamic [41, 42]. As the wound closes and evolves into a scar, apoptosis of the activated fibroblasts (myofibroblasts) leads to a significant decrease in their numbers [43]. The inability of myofibroblasts to undergo apoptosis is the driving factor in the development of fibrotic diseases and contributes for the maintenance of other pathological conditions, such as chronic inflammation [42].

In oncologic settings, fibroblasts are frequent components of the TMEN and play an important role at all stages of cancer progression through their phenotypic plasticity and ability to secrete a wide range of signaling molecules. Next, we emphasize how these cells play a key role in generating tumor-promoting microenvironments in GI cancers.

### 3.1. Role of CAF in Cancer Initiation and Growth

Multiple studies have highlighted the important role of CAFs in the process of cancer initiation and progression. For example, the abundance of myofibroblasts in CRC-associated stroma is predictive of postsurgery disease recurrence [44]. It has also been suggested that the loss of transforming growth factor- (TGF-) *β* inhibitory effect leads to the activation of hepatocyte growth factor- (HGF-) mediated cell-cycle regulation and stimulation of epithelial proliferation, promoting invasive squamous cell carcinoma of the forestomach in the Tgfbr2^*fspKO*^ knockout mice [45]. In addition, the conditional knockout of the TGF-*β* type II receptor gene in mouse fibroblast-specific protein 1- (FSP1-) positive fibroblasts revealed that TGF-*β* signaling modulates the proliferation and oncogenic potential of epithelial cells [45].

Recently, it has also been demonstrated that CAFs-secreted HGF regulates liver tumor-initiating cell plasticity through the activation of Tyrosine-Protein Kinase Met/Fos-Related Antigen 1/Hairy and Enhancer of Split-Related Protein 1 (c-Met/FRA1/HEY1) signaling. The activation of this signaling pathway was associated with fibrosis-dependent development in HCC *in vivo* [46]. CAFs-derived HGF was also shown to promote a stemness phenotype in CRC cells [47]. In another study, the deletion of Liver Kinase B1 (Lkb1) gene in stromal fibroblasts resulted in penetrant polyposis in mice, underscoring the involvement of these cells in the tumorigenesis of GI Peutz–Jeghers syndrome. Further analysis revealed that Lkb1 loss induces interleukin- (IL-) 11 expression in gastric fibroblasts and subsequent activation of the Janus Kinase/Signal Transducer and Activator of Transcription 3 (JAK/STAT3) pathway in tumor epithelia, promoting GI tumorigenesis [48].

Emerging data also suggest the switch from normal quiescent fibroblasts into an activated phenotype through epigenetic modifications [49–51]. *Helicobacter pylori* infection, one of the major causes of GC, was shown to induce the secretion of Prostaglandin E2 (PGE2) by gastric epithelial cells. The stromal PGE2 silenced miR-149 through hypermethylation, removing the suppression of its target genes, IL6 and PGE2 receptor 2. This led to elevated IL6 levels that stimulated the stem-like properties of GC cells [49].

### 3.2. Role of CAF in EMT, Extracellular Matrix Remodeling, and Metastasis

CAFs-mediated signaling also participates in the acquisition and maintenance of cancer cell stemness. One of the common concepts associated with metastasis initiation is epithelial-to-mesenchymal transition (EMT), that is, the process by which cells lose their epithelial characteristics (such as cell-to-cell adhesion and planar and apical polarity) to acquire mesenchymal features (such as motility and invasiveness) [52]. Paracrine signaling through TGF-*β* between CAFs and cancer cells leads to EMT-driven gain of stemness and metastasis initiation [53, 54]. In fact, targeting CAFs with curcumin reverted the EMT phenotypes of PDAC cells blocking their migration and metastasis [55]. In HCC, myofibroblasts can induce EMT in a TGF-*β*/platelet-derived growth factor- (PDGF-) dependent manner [56]. Likewise, IL-6 produced by fibroblasts can also activate JAK2/STAT3 signaling in the GC cells promoting their migration and EMT [57]. The miRNA 320a can also affect EMT by decreasing PBX Homebox 3 (PBX3), Extracellular Signal-Regulated Kinase 1 and 2 (ERK1/2) signaling, and N-cadherin expression, and simultaneously increase E-cadherin. When transferred from CAFs to HCC tumor cells via exosomes, this miRNA can inhibit tumor proliferation, migration, invasion, and metastasis. Interestingly, CAF-derived exosomes from HCC patients contain reduced levels of miR-320a, showing how the reduction of an antitumor factor in these vesicles can affect metastasis [58]. Cancer cell-derived exosomes can also reprogram normal adjacent fibroblasts into CAFs. For example, a recent study showed that exosomes derived from early- or late-stage CRC cell lines induce the activation of quiescent fibroblasts into distinct functional subtypes [59]. Specifically, the activation mediated by late-stage cancer exosomes resulted in a proinvasive profile, while fibroblasts activated by early-stage cancer exosomes presented a pro‐proliferative and proangiogenic phenotype [59].

Fibroblasts can also remodel the microenvironment and lay the tracks for cancer cell invasion through the surrounding ECM and stromal cell layers [60–62]. For instance, CAF-derived transgelin (TAGLN) induces MMP2 expression and promotes migration and invasion of GC cells [63], while CAF-derived fibroblast activation protein (FAP) remodels the ECM and promotes PDAC cell invasion [64]. In HCC, CAFs secrete different cytokines that activate the hedgehog (C-C motif chemokine 2 (CCL2) and 5 (CCL5) and TGF-*β* (CCL7 and C-X-C motif ligand (CXCL) 16) pathways in HCC cells, inducing their migration and invasion *in vitro* and metastasis *in vivo* [65]. In addition, Granulocyte-Macrophage Colony-Stimulating Factor (GM-CSF) secreted by cancer-associated MSCs, a subpopulation of CAFs isolated from human PDAC, induced proliferation, invasion, and transendothelial migration of PDAC cells [66].

Colonization is probably the most limiting of all metastasis steps and the microenvironment at the distant sites needs to be favorable for this to happen. Paget's seed and soil theory back in 1889 was the first to suggest that metastasis to a certain organ is not random but depends on interactions with its microenvironment and that cancer cells will seed only in fertile soils [67]. The concept of “pre-metastatic niche” was introduced later in 2001 by Kaplan et al., where they showed that BM-derived cells can form clusters that home to tumor-specific premetastatic sites [67, 68]. In line with this theory, metastatic cancer cells are capable of bringing their own “soil” to the metastatic site in order to facilitate their colonization [69]. A good example is the case of IL11 production by TGF-*β*-stimulated CAFs, which activate Glycoprotein 130 (GP130)/STAT3 signaling in CRC cells conferring them the survival advantage for efficient organ colonization [70].

### 3.3. Role of CAF in Angiogenesis

CAFs tailor tumor growth and progression not only by influencing tumor cells but also by indirectly affecting other stromal cells and regulating angiogenesis, inflammation and immune modulation [10]. CAFs are capable of promoting angiogenesis by secreting VEGF and Stromal Cell-Derived Factor 1 (SDF-1) [71, 72]. For example, in a mouse model of GC, *α*-Smooth Muscle Actin- (*α*-SMA-) positive fibroblasts were the main producers of VEGF. Activation of these fibroblasts was stimulated by GC cells and shown to increase tube formation by endothelial cells *in vitro* [73]. CAFs are also a source of IL6 in CRC, which in turn can increase VEGF secretion by adjacent fibroblasts and induce tumor angiogenesis in xenografted cancer cells [74]. Pancreatic fibroblasts, also known as pancreatic stellate cells (PSCs), express several proangiogenic regulators such as VEGF receptors, angiopoietin-1, and Tie-2 and produce VEGF in response to hypoxia. Conditioned medium from hypoxia-induced PSCs was able to increase proliferation, migration, and angiogenesis of Human Umbilical Vein Endothelial Cells (HUVECs) both *in vitro* and *in vivo* [75]. In a similar line, hepatic stellate cells (one of the main players in HCC) can produce VEGF and angiopoietin-1 in hypoxic conditions and induce angiogenesis [76, 77].

## 4. Endothelial and Perivascular Cells

### 4.1. Role of Endothelial and Perivascular Cells in Tumor Growth

The angiogenic switch is a hallmark of cancer that allows for tumor growth by providing nutrients and oxygen and removing cellular wastes [10]. The establishment of new blood vessels is a crucial step for tumor progression, but the endothelial and perivascular cells that constitute these blood vessels are not just mere bystanders in the game. Endothelial cells (ECs) can promote a cancer stem cell phenotype in human CRC *in vitro* and in cocultured CRC cells ex vivo [78]. Lu et al. showed that CRC stemness is induced through paracrine activation of *Notch* signaling, whereby membrane-bound Jagged-1 on ECs is cleaved by ADAM metalloproteinase domain 17 (ADAM17), releasing a truncated soluble fragment that binds Notch on CRC cells. Importantly, both primary and chemotherapy-naïve liver metastatic CRC liver showed CD133^+^ epithelial cells located in the proximity to perivascular regions, further supporting an ECs-mediated role in the CRC stem cell phenotype in clinical specimens [78]. A subsequent study found that ECs contributed to chemoresistance in CRC cells via serine/threonine-protein kinase- (AKT-)mediated induction of Nanog Homeobox Retrogene P8 (NANOGP8) [79]. Likewise, in an *in vitro* model of Hepatitis B Virus- (HBV-) induced HCC, increased levels of TGF-*β* in the conditioned medium of HUVECs boosted the expression of mesenchymal markers, including CD133, and promoted an aggressive phenotype in stimulated Hepatitis B-X Protein- (HBx-) infected hepatoma cells [80]. Immunization of mice with glutaraldehyde-fixed HUVECs resulted in reduced expression of angiogenesis-related antigens (VEGF-2 and vascular endothelial- (VE-) cadherin), suppression of angiogenesis, and smaller esophageal squamous carcinoma (ESC) tumors [81]. This has prompted the evaluation of HUVEC vaccines promoting tumor autoimmune response targeting angiogenesis in pilot trials involving patients with metastatic CRC [82].

Lymphatic endothelial cells (LECs) are also important players in the growth of GI cancers. Expression of Growth Differentiation Factor 11 (GDF11) was increased in CRC patients and positively correlated with tumor grade. GDF11 released in LEC-derived exosomes was also identified as a key modulator of CRC growth *in vitro* and *in vivo* [83]. Moreover, increased proliferation and invasive ability of ESC cells *in vitro* has been demonstrated upon stimulation with conditioned medium from ESC-related lymphatic microvessel endothelial cells. This interaction leads to the upregulation of MMP9 expression and downregulation of Tissue Inhibitor of Metalloproteinases 2 (TIMP-2) expression in poorly differentiated ESC cells and promotes both lymphangiogenesis and growth of these cells *in vivo* [84]. Lymphatic endothelial cells also have an immunoregulatory function in GC by inhibiting the production of IL2, IL10, and IFN-*γ* cytokines in CD4^+^ T cells. Coculturing GC cells with both LECs and CD4^+^ T cells resulted in the upregulation of Programmed Death-Ligand 1 (PD-L1) and Indoleamine 2,3-Dioxygenase (IDO) mRNA expression. This suggests a possible mechanism of cancer immune tolerance and metastatic spread through the lymphatic vessels in GC [85].

Additionally, the development of diffuse-type GC depends on the inflammation mediated by CXCL12^+^ ECs and C-X-C motif receptor (CXCR) 4^+^ gastric innate lymphoid cells (ILCs) that form the perivascular gastric stem cell niche. Endothelial CXCL12 plays a central role recruiting Wnt Family Member 5A- (Wnt5a-) producing CXCR4^+^ ILCs to the stomach, which in turn activates Ras Homolog Family Member A (RhoA), inhibits anoikis in the E-cadherin-depleted cells and promotes diffuse-type GC growth [86].

### 4.2. Endothelial and Perivascular Cells Role in Tumor Metastasis

Blood vessels are also a gateway for distant metastasis. The lack of vascular maturation in newborn vessels facilitates cancer cell penetration and promotes distant metastasis. Immunohistochemical analysis of tissue samples from CRC patients revealed a significant correlation between lower microvessel pericyte coverage with increased hematogenous metastasis and poorer survival [87].

The importance of these vascular networks is underscored by the fact that cancer cells undergoing EMT are able to assume the identity and role of pericytes to stabilize the tumor vasculature and improve the vascular support for tumor growth [88]. Shenoy et al. found that the majority of cancer cells undergoing EMT were located preferentially in the perivascular space and were closely associated with ECs and in line with the blood vessels in tumor xenografts. It was further demonstrated that these cells expressed pericyte markers and interacted with ECs, stretching alongside Human Microvascular Endothelial Cells (HMVECs) and exhibiting tight adhesion to EC tubes in a coculture assay *in vitro* [88]. This phenomenon, known as vasculogenic mimicry (VM), whereby cancer cells form *de novo* vascular networks without the involvement of ECs, represents an alternative paradigm of tumor perfusion in HCC and GC [89–91].

Exosomes are also key mediators in this setting, as they mediate vascular permeability and angiogenesis. Zeng et al. found that CRC-derived exosomal miR-25-3p can be transferred to ECs, where it targets and silences Krüppel-Like Factor 2 (KLF2) and 4 (KLF4). *In vivo*, exosomal miR-25-3p elicited vascular leakiness and promoted CRC metastasis [92]. Exosome-mediated remodeling of the lymphatic network in sentinel lymph nodes was also shown to promote CRC metastasis. The uptake of CT26-derived exosomes by macrophages induced the release of VEGF-C, mediated by exosomal Interferon Regulatory Factor 2 (IRF-2), and promoted lymphangiogenesis in sentinel lymph nodes, which facilitated the development of CRC metastasis [93]. Upregulated secretion of CXCL1 by LECs can also promote migration, invasion, and adhesion of GC cells through the activation of integrin *β*1- (Focal Adhesion Kinase) FAK-AKT signaling. Activation of the latter induced the expression of MMP2 and MMP9 and increased lymph node metastasis in an animal model of GC [94].

When cancer cells enter the systemic circulation, they get exposed to the harsh circulating conditions. Together with the absence of cell/ECM interaction, apoptosis signaling is triggered and cancer cells undergo rapid anoikis [95]. A recent study in head and neck carcinoma showed that cancer cells bound to Bcl-2 overexpressing ECs (EC-Bcl-2) via E-selectin presented significantly higher anoikis resistance. Additionally, mice coinjected with squamous cell carcinoma cells and EC-Bcl-2 displayed significantly higher lung metastasis [96]. The described chaperoning role of ECs could, in principle, also occur in GI cancers, as increased levels of circulating ECs have also been observed in patients with colon, gastric, and esophagus cancers [97].

## 5. Tumor-Infiltrated Immune Cells

Infiltrating immune cells from lymphocytic and myeloid origin are also constituents of the TMEN [10]. Lymphocytes are composed by three main lineages that originate from a common precursor identified in the BM: natural killer (NK) cells, T cells, and B cells. T cells are subject to a final lineage decision in the thymus to form mature CD4 (helper) and CD8 (cytotoxic) T cells [98]. NK and CD8 T cells can rapidly degranulate and secrete IFN-*γ* following antigen receptor triggering, which is particularly important in antitumor responses [99]. T regulatory Cells (TRegs) can contribute to homeostasis by inducing immunotolerance and control of autoimmunity. These cells are derived from the thymus, express CD4, CD25, and Forkhead Box P3 (FOXP3, murine Foxp3) and are capable of inhibiting immune responses mediated by CD4^+^CD25^−^ and CD8^+^ T cells [100–102].

Myeloid cells comprise polymorphonuclear cells or granulocytes, dendritic cells (DCs), monocytes, and macrophages (extravasated blood monocytes) [103, 104]. Macrophages can display a broad spectrum of activation and polarization states [105, 106]. However, in more general and simplistic models, they are frequently classified as M1, that takes part in type I T helper (Th1) cell responses when stimulated by IFN-*γ* and is characterized by release of radical oxygen species (ROS), nitric oxide (NO), and proinflammatory cytokines such as TNF-*α* and IL12; M2, involved in type II T helper (Th2) cell responses and identified by expression of arginase and release of anti-inflammatory cytokines such as IL10 when stimulated by IL4 and IL13 [107].

Myeloid-derived suppressor cells (MDSC) comprise another population of myeloid progenitor and immature myeloid cells endowed with the ability to inhibit T-cell responses [108–110]. DCs were also reported as activators of specific T cells during inflammatory responses and play a central role in protection against infection and malignancy [111, 112]. DCs can also display multiple profiles. Monocyte-derived DCs can perform marked tumor antigen uptake. cDC1s can activate CD8^+^ T cells, while cDC2s can reprogram protumoral macrophages when injected in mouse models. Importantly, the analysis of tumor biopsies from colorectal cancer patients revealed the presence of all the three abovementioned subsets of tumor-associated DCs [113]. For these reasons, myeloid cells play a pivotal role in the regulation of immune cell responses.

Tumor inflammation has a paradoxical role in promoting tumor growth and progression [10]. Some reports show the association between unresolved infection, chronic inflammation, and tumor initiation. Examples are the relationships between infection by *Helicobater pylori* and GC, chronic pancreatitis and PDAC, and ulcerative colitis and CRC [114–117]. In this section, we describe how immune cells contribute to TMENs in GI cancers.

### 5.1. Immune Surveillance Evasion

Although increased infiltration of tumors by immune cells has long been thought to be a consequence of failed attempts to eliminate cancer cells, recent studies show that cancer cells can frequently evade immune responses [10]. An important mechanism of immune evasion involves PD-L1/Programmed Death-1 (PD-1) that has been linked to T-cell apoptosis [118]. For example, HCC-derived IL-10 can increase expression of PD-L1 by Kupffer cells, which in turn can decrease the antitumor function and proliferation of CD8^+^PD-1^+^ cells [119]. In the same line, PD-L1^+^ monocytes infiltrates can suppress antitumor T-cell responses and contribute to tumor growth *in vivo* [120]. In both cases, PD-L1 correlated with poor survival in HCC patients and could be targeted by anti-PD-L1 antibodies [119, 120]. CXCL12 produced by FAP^+^CAFs has been linked to immune evasion in PDAC, and targeting this cytokine can synergize with anti-PD-L1 immunotherapy in PDAC [121, 122].

In two complementary studies, Wang et al. and Kortylewski et al. demonstrated how the constitutive activation of Stat-3 in cancer and hematopoietic-derived tumor infiltrating cells could inhibit the maturation of DCs, leading to a defective antitumor immune response. In fact, Stat-3 inhibition enhanced the antitumor function of T and NK cells, DCs, and innate immunity against tumors [123, 124]. Furthermore, in a small cohort of patients and healthy control subjects, Yanagimoto et al. have shown that the numbers and function of DCs were reduced in PDAC patients [125].

PDAC and CRC cells can also evade immune response by expressing an apoptosis-mediating surface antigen FAS (Fas) receptor, which enables these cells to resist Fas-mediated apoptosis and, at the same time, to increase the expression of Fas ligand (FasL), which mediated the killing of T cells in coculture assays [126–128]. The upregulation of FasL was also demonstrated in GC together with downregulation of caspase-3 as an immune escape mechanism [129, 130]. Another strategy by which PDAC cells could escape immune response was through the upregulation of IDO [131], a tryptophan-degrading enzyme that induces an anergic state in T cells through tryptophan starvation [132]. In addition, the presence of Th2 lymphocytes also correlates with reduced PDAC patient survival. Thymic Stromal Lymphopoietin (TSLP), which induces Th2 polarization, was found to be secreted by CAFs after stimulation by TNF-*α* and IL1*β* [133].

Immunosuppressive cells such as MDSC and TRegs are elevated in patients with PDAC, esophageal, and GC when compared with controls, being considered an independent prognostic factor for survival in all these three cancers [134]. In fact, by studying a small cohort of patients, Porembka et al. have demonstrated that human PDAC show an increased infiltrate of MDSC when compared with normal pancreatic tissue. Moreover, depletion of these cells in an animal model of PDAC resulted in delayed tumor growth [135]. MDSCs were also increased in HCC patients and could induce the formation of TReg populations, suggesting this as one of the mechanisms responsible for immunosuppression in HCC [136]. Increased populations of TRegs were also found in the blood of patients with gastric and esophageal cancers [137]. In addition, TGF-*β*1 produced by GC cells was shown to induce TReg development from CD4^+^CD25^−^T cells, and high levels of this factor correlated with elevated TReg numbers in GC patients [138]. Interestingly, Mizukami et al. suggested that the localization pattern rather than the numbers of TRegs might have a higher impact in survival of GC patients. They found that a diffuse pattern of TRegs accounts for poorer survival than peritumoral localization of these cells [139]. TRegs of HCC patients were also capable of impairing the function of CD8^+^ T cells by decreasing their proliferation, activation, degranulation, and production of enzymes such as granzymes A and B and perforin when stimulated. Not surprisingly, TRegs were associated with higher mortality in these patients [140]. Both CRC- and HCC-associated fibroblasts were also shown to impair NK-cell antitumor cytotoxicity by releasing molecules such as PGE2 [141, 142].

### 5.2. Immune Cells Role in EMT, Invasion. and Metastasis

In addition to immune surveillance evasion, infiltrated immune cells also promote invasive phenotypes in cancer cells through EMT. For example, PDAC cells increase the conversion of blood monocytes into monocytic MDSC, which in turn act to promote EMT features in these cancer cells [143]. Using *in vitro* coculture studies, Liu et al. have shown that the promotion of the M2 phenotype in macrophages induces the expression of mesenchymal markers in PDAC cell lines *in vitro* and that this effect was dependent on Toll-Like Receptor 4 (TLR4) and IL10 levels [144]. M2 macrophages were also capable of inducing GC invasiveness via activation of the *β*-catenin pathway [145].

Kim et al. suggested that myofibroblasts can induce the differentiation of myeloid cells into S100A8/9-expressing MDSC and M2 macrophages in CRC by secreting IL-6 and IL-8 [146]. In a mouse model of esophageal cancer, recruitment of MDSC was correlated with IL-6 levels and tumor invasiveness, IL-6 being shown to induce the MDSCs. In fact, IL-6 and MDSC levels predicted prognosis in patients with esophageal cancer [147]. In addition, CAFs and M2 macrophage markers predict clinical outcome in CRC, their expression being inversely correlated with survival [148]. Similarly, CAFs isolated from PDAC patients promoted M2 macrophage polarization that in turn promoted the proliferation of PDAC cells *in vitro* and tumor growth and invasion *in vivo* [149]. Polarized CD163^+^ (M2) macrophages were also correlated with increased angiogenesis, CXCL12 expression, and tumor progression in GC [150].

The recruitment of immune cells to secondary metastatic sites, and their role in promoting a receptive soil for metastatic growth, has also been the focus of some recent studies. For example, PDAC-derived exosomes containing macrophage migration inhibitory factor- (MIF-) induced TGF-*β*1 production by Kupffer cells, which induced *α*-SMA and fibronectin expression by hepatic stellate cells. This supported the influx of BM-derived monocytes, which constituted a liver TMEN supportive of PDAC metastasis [151]. In fact, PDAC liver metastasis depends on the early recruitment of granulin-secreting inflammatory monocytes to this organ. Granulin secretion by metastasis-associated macrophages activates resident hepatic stellate cells into myofibroblasts, which in turn secrete periostin, resulting in a fibrotic microenvironment that sustains metastatic tumor growth [152]. Seubert et al. also demonstrated an increased liver susceptibility towards metastasis through SDF-1-mediated recruitment of neutrophils to the liver. In this study, systemic TIMP-1, which was previously thought to suppress tumor metastasis, was instead driving the increased levels of hepatic neutrophil chemoattractant SDF-1 [153].

## 6. Stromal Signatures as Prognostic Tools

Based on the significance of stromal cells in tumor growth and progression, much effort has been done on the identification of stromal signatures of cancer prognosis. For example, tumor lymphocyte infiltrates (TIL), such as CD4^+^/CD8^+^ T and NK^+^ cells, have been generally associated with a good prognosis. On the other hand, infiltration by TRegs, MDSC, M2 macrophages, and CAFs has been seen as a sign of disease progression and poor prognosis, as listed in Table 1.

However, conflicting evidence has shown that increased infiltration of gastric and gastroesophageal tumors by CD8^+^ T cells was actually associated with a worse prognosis. In fact, patients with high CD8 infiltration also presented PD-L1 expression, which was linked to immune resistance [160]. On the other hand, microsatellite unstable GC patients with high CD163^+^ (M2) macrophages, FOXP3^+^, and CD8^+^ TILs where those with the highest survival advantage [182]. In CRC, as opposite to other cancer types, FOXP3^+^ TRegs were associated with good prognosis [170, 171]. These are examples on how immune cell signatures are context-dependent and how the complexity of cell interactions and soluble factors released in the TMEN can tip the balance in opposite directions.

Given the diversity of switch mechanisms driving CAFs activation, one can expect to have CAFs with different activated phenotypes in the tumor stroma. Another question is whether all CAFs are in an activated state. Fibroblast plasticity and intratumoral heterogeneity results in an array of CAF signatures associated with different tumor types [184]. Several proteins have been used as markers for the identification of CAFs. Some of the most commonly used biomarkers include PDGFR*α*/*β*, *α*-SMA, and FAP. In addition, FSP1 has been suggested as a marker of fibroblasts in a quiescent state [185, 186]. Other proteins, such as vimentin, desmin, discoidin domain receptor 2 (DDR2), and podoplanin, have also been used in the identification of CAFs [185]. However, it is important to highlight that these proteins are also expressed by other cell types, and the lack of consistent and specific fibroblast molecular markers has been an important limiting factor so far [185]. Opposing actions of CAFs expressing the same protein marker can also be observed in a context-dependent way in TMENs. For example, while in CRC-associated CAFs, podoplanin was correlated with less aggressive tumors and a favorable prognosis [187, 188], its expression by CAFs in lung, breast, esophageal, and PDAC has been associated with an unfavorable prognosis [189–192]. In addition, PDAC patients with fewer myofibroblasts in the tumors had reduced survival, possibly by suppression of the immune surveillance due to increased levels of TRegs [193]. Therefore, one should be cautious when identifying CAFs and extrapolating their role in different tumors based on the analysis of the aforementioned biomarkers. It is increasingly evident that CAFs of tumors from different etiologies present different molecular biomarkers or combination of biomarkers [194].

The full spectrum of this phenotypic diversity and their functional implications in tumor growth, progression, or even therapy resistance mechanisms are yet to be fully understood. However, defining specific tumor-associated immune and CAF signatures might become a valuable prognostic tool and drive the advancement of new therapeutic strategies.

## 7. Impact of Stroma in Resistance to Therapies

During cancer progression, tumors become more heterogeneous due to a generation of genetically distinct tumor-cell subpopulations and to modifications in TMEN components. In this section, we will describe how tumor heterogeneity, combined with the high plasticity of tumor-associated cells, can influence resistance to therapies in GI cancers.

### 7.1. Desmoplasia and Tumor Resistance

A desmoplastic reaction, characterized by the formation of a dense fibrosis and increased remodeling and deposition of ECM components, is closely associated to a poor outcome in PDAC and CRC patients [195, 196]. One of the main components of the ECM is hyaluronic acid (HA), which is a high molecular mass polysaccharide. PDAC can express HA into stroma and in peritumoral connective tissue and thus impair vascularity and the delivery of chemotherapeutic drugs into tumors. In fact, gemcitabine-resistant PDAC from patients with resectable tumors showed upregulation in gene pathways related to stroma-ECM receptor interaction, focal adhesion, cell communications, gap junction, and cell adhesion molecules [197]. Enzymatic degradation of HA results in reduction of interstitial flow pressure, reexpands the microvasculature in PDAC [198], and increases the delivery of doxorubicin and gemcitabine in a mouse model of PDAC [199].

The Sonic hedgehog (Shh) pathway also promotes desmoplasia in PDAC, and its inhibition improves delivery of chemotherapy [200]. However, genetic inhibition of the Shh pathway results in more aggressive tumors in a PDAC model [201] and accelerates progression of KRAS-driven PDAC. Inhibiting VEGF receptor (VEGFR) in Shh-deficient mice increased survival and impaired tumor progression [202], suggesting that combinatory approaches could be more effective to overcome tumor resistance.

CAF heterogeneity might be responsible, at least in part, for the protumorigenic and antitumorigenic effects in cancer resistance. For example, PDAC presents a subpopulation with high expression of *α*-SMA adjacent to neoplastic cells, and another with low expression of *α*-SMA that locates distantly and secretes inflammatory mediators as IL-6 [203]. Intriguingly, depletion of CAFs based on their *α*-SMA expression can induce immunodepression and accelerate pancreas cancer progression [193], leading to resistance to chemotherapies. These pieces of evidence indicate that new therapeutic approaches should consider these different subpopulations when looking for effective antitumor therapies directed to CAF.

Chemotherapy can also affect stromal cells, which in turn can promote cancer resistance. A hypoxic TMEN can lead to a metabolic shift based on aerobic glycolysis and lactate production by tumor cells, leading to a low extracellular pH, which is a common feature found in solid tumors. Moreover, chemotherapy-treated CAFs change the expression of metabolic enzymes, leading to increased aerobic glycolysis and autophagy and increased energy production [204]. A recent study showed that drugs targeting mutated K-Ras force cancer cells to get energy thought autophagy in PDAC [205].

Low expression of caveolin-1 in stroma is a marker of autophagy, which occurs via oxidative stress followed by an increase in HIF-1*α* and NF-kappa B expression [206]. High level of HIF-1*α* in CAFs is related to an elevated lactate efflux and lower extracellular pH [207]. This acid microenvironment drives EMT, protecting PDAC cells from gemcitabine-induced cell death in a mechanism that involves expression of drug transporters [208]. Moreover, gemcitabine upregulates CXCR4 expression in PDAC cells and promotes their invasiveness through a reactive oxygen species-dependent mechanism [209].

### 7.2. Soluble Factors and Exosomes Roles in Tumor Resistance

Stromal cells produce soluble factors that play a key role in chemoresistance (Table 2). For example, expression of TGF-*β*1 by CAFs is frequently present in patients treated with chemoradiotherapy, its inhibition being linked to enhanced chemosensitivity of ESC cells [214]. CAFs can also release IL6, which activates the JAK-1/STAT3 signaling pathway and contributes to chemoresistance of GC cells to 5-fluorouracil (5-FU) [217]. IL-6 secreted by CAFs also plays a role in chemoresistance of ESC cells by upregulating CXCR7. In fact, ESC patients with high expression of CXCR7 and IL-6 presented worse overall survival upon receiving cisplatin after surgery [214].

Tumor-associated macrophages (TAM) release IL6, which activates the IL-6 receptor (IL6R)/STAT3 pathway in CRC cells. STAT3 inhibits the tumor suppressor miR-204-5p, leading to chemoresistance to 5-FU and to oxaliplatin [218]. This suggests that IL6 receptor inhibition in combination with chemotherapy could serve as a suitable strategy to improve chemotherapeutic efficacy through inhibition of the communication between stromal and GC cells [217]. Another example involves cisplatin resistance in GC cells by TAM-derived exosomes containing miR-21 [216]. Exosomal transfer of miR-21 led to downregulation of PTEN and activation of AKT, which resulted in less apoptosis and increased survival in GC cells treated with cisplatin [216].

Moreover, crosstalk between TAM and tumor infiltrating cells through STAT3 can improve chemotherapeutic efficacy by repressing antitumoral CD8^+^ T-lymphocyte activity [219].

Treatment failure can also result, at least in part, from the increase in exosome release by stromal cells. For instance, gemcitabine treatment increases fibroblast-derived exosomes containing Snail and miR146a, a Snail target, which induce resistance to chemotherapies in PDAC [210] and promote metastasis and chemotherapy resistance by enhancing cell stemness and EMT in CRC cells [220]. Upon exposure to oxaliplatin, CAFs may release exosomes containing long noncoding RNA (lncRNA) H19 to cancer cells, which has competing endogenous RNA potential for miR-141, a tumor suppressor miRNA that targets *β*-catenin and suppresses the Wnt/*β*-catenin pathway. In this way, lncRNA H19 promotes stemness of cancer stem cells and oxaliplatin resistance of CRC [211]. Similarly, exosomes secreted by gemcitabine-treated CAFs promote proliferation and gemcitabine resistance of PDAC cells by increasing Snail expression [210]. Pancreas-derived mesenchymal stromal cells treated with paclitaxel release exosomes containing paclitaxel which inhibit proliferation of PDAC [221]. Moreover, the intracellular and extracellular expression levels of miR-145 and -34a in CRC cells were associated with 5-FU resistance [212]. The resistance was in part due to the enhanced secretion of these antioncomirs in exosomes produced by resistant CRC cells after 5-FU exposure. This led to decreased intracellular levels of the antioncomirs and sustaining proliferation [212]. 5-FU and oxaliplatin treatment can also induce CAFs to release soluble factors that are taken up by CRC cells, promoting drug resistance through AKT, P38, and survivin translocation [215]. In addition, Snail-expressing fibroblasts can secrete CCL1 and contribute to 5-FU and paclitaxel chemoresistance in CRC [222]. Similarly, increased Snail expression in PDAC cells is correlated with gemcitabine resistance [223].

## 8. Therapies Targeting Stromal Microenvironment

### 8.1. Extracellular Matrix Components

In 2003, the first clinical trial of a humanized monoclonal antibody directed to human FAP, sibrotuzumab, was found clinically safe in patients with advanced solid cancers [224]. However, it showed limited clinical response in a phase II trial in patients with CRC [225]. Inspite of promising preclinical findings, therapy strategies targeting CAFs have repeatedly faced obstacles. As we pointed out above, depletion of *α*-SMA-expressing CAFs can accelerate pancreas cancer progression [193]. Thus, depleting CAFs based on their expression of FAP or *α*-SMA might not be effective, since other stromal cell types can also express these markers.

Regarding ECM remodeling, Lysyl Oxidase-like 2 (LOXL2) is upregulated in tumor-associated stroma of PDAC, ESC, and HCC [226, 227]. Simtuzumab, an antibody that inhibits LOXL2, blocks the desmoplastic reaction in CRCs in vitro [226]. However, phase II clinical trials of simtuzumab in combination with gemcitabine or FOLFIRI (folinic acid, 5-FU, irinotecan) did not improve the clinical outcome in PDAC or in CRC patients, respectively [228, 229].

Different approaches that inhibit desmoplasia in solid cancers can inhibit tumor growth and improve vascular perfusion and drug delivery. Losartan (an angiotensin I inhibitor) is an antihypertensive drug that reduces collagen and hyaluronan production by CAF through downregulation of the fibrotic signals TGF-*β*1, Cellular Communication Network Factor 2 (CCN2), and Protein Effector of Transcription 1 (ET-1) [230]. In fact, epidemiological studies demonstrated that gastroesophageal cancer patients presented a moderately reduced cancer-specific mortality amongst users of angiotensin receptor blockers [231]. Based on preclinical studies, a phase II study targeting TGF-*β*1 by using losartan in combination to FOLFIRINOX (folinic acid, 5-FU, irinotecan, oxaliplatin) in locally advanced PDAC is ongoing with an estimative to be concluded by 2025 (Table 3). Another approach is to inhibit Shh signaling, which drives stromal desmoplasia, by activating the ligand for smoothened (SMO) in CAFs [237]. The SMO inhibitor (IPI-926) reduced the abundance of myofibroblasts in the stroma in PDAC and increased tumor vasculature as well as intratumoral gemcitabine uptake. However, a phase Ib/II clinical trial using IPI-926 in combination with gemcitabine in metastatic PDAC did not show benefits in clinical outcome. Indeed, some patients receiving IPI-926 had a shorter median survival time compared with the placebo group [200].

The tumor stroma can also play an important role in restraining tumor growth, mainly due to the heterogeneous population of fibroblast present in PDAC. Preclinical studies identified a CAF subpopulation expressing high amounts of *α*-SMA close to tumor cells and CAF subpopulations expressing low *α*-SMA and secreting IL-6 which could be responsible for the aggressiveness of PDAC [193, 201]. A phase Ib study using enzymatic ablation of hyaluronan by PEGPH20, a PEGylated recombinant hyaluronidase, in combination with gemcitabine showed a potential therapeutic benefit, especially in patients with high expression of HA [232]. In fact, a phase II clinical trial using PEGPH20 in association with gemcitabine and nab-paclitaxel showed improvement in the progression-free survival of PDAC patients [233] and it is now being evaluated in a phase III trial (Table 3). However, PEGPH20 in association with a modified FOLFIRINOX regimen presented high toxicity when compared with FOLFIRINOX alone [238].

Other strategies to inhibit FAP in PDAC showed promising results in preclinical studies [121, 239, 240]. For example, anti-FAP CAR T cells can deplete FAP^+^ cells in PDAC and decrease tumor growth through promotion of antitumor immunity [241]. Recently, a preclinical trial using a DNA vaccine against FAP synergized with anticancer immune therapy targeting Prostate Membrane Antigen (PMSA) in tumor-bearing mice model for prostate cancer [242]. This result suggests that therapies which target both stroma components and tumor cells might be effective for tumors expressing high amounts of FAP, such as CRC and PDAC.

### 8.2. Immune System

PD-1 is expressed on a large proportion of TILs from many different cancer types, while its ligand, PD-L1, is mainly expressed in antigen-presenting cells and tumor cells [243]. Since tumors can escape the T cell immune response by expressing these molecules, the blockade of this pathway has emerged as a promising anticancer strategy. This approach also showed good results as second and third line of chemotherapy in gastro-esophageal cancer [244, 245]. A clinical study evaluating nivolumab (an antibody against PD-1) monotherapy in heavily pretreated patients with advanced gastric or gastro-esophageal junction cancer showed an increased 12-month overall survival rate compared to the placebo group [244]. In another trial, both objective and complete responses were observed in patients with gastro-esophageal cancer treated with pembrolizumab (an antibody against PD-1) monotherapy, irrespective of PD-L1 tumor expression. Nonetheless, pembrolizumab conferred longer response duration in those patients with PD-L1–positive tumors [245].

CTL4-A (cytotoxic T-lymphocyte antigen 4) signaling diminishes immune response against tumor cells and the use of antibodies against CTL4-A was effective in treating tumors as melanomas [246]. However, clinical trials in PDAC using monotherapies with CTLA-4 or PD-1 inhibitors presented low response rates [247, 248], with the exception of the PDAC patient subpopulation with microsatelite instability [249]. Although the response rates from these studies remain discouraging, they could be improved by combinatory therapies. A phase II study with Nivolumab in association with Ipilimumab (an antibody against CTLA-4) in patients presenting upper GI cancers is ongoing (see Table 3). The first trials using nivolumab and pembrolizumab in HCC were encouraging [250, 251]. However, pembrolizumab as second line of treatment did not meet its coprimary endpoints of overall survival and progression-free survival [252]. A phase III trial with nivolumab in first line treatment is currently underway. Unfortunately, when a better selection of patients based on molecular characteristics from the tumor or on its etiology was performed, the data was inconclusive [250, 251]. Another study showed that a therapy targeting FAP^+^ cells that express CXCL12 synergized with anti-PD-L1 immunotherapy in PDAC [121], and inhibition of its receptor, CXCR4, in sorafenib-treated HCC facilitates anti-PDL-1 immunotherapy [253]. In addition, CXCR4 inhibition increased PD-1 therapy response by inducing mobilization of CD8^+^ T cells in PDAC [254]. Together, these studies demonstrate important systemic components that might play a role in the clinical outcome and explain, in part, the heterogeneous therapeutic response normally found in these clinical trials.

Natural Killer Cells Antigen 94 (CD94/NGK2a) is the main HLA-E receptor which mediates an inhibitory effect on CD8^+^ CTL and NK cells, promoting immune evasion in CRC [255]. In fact, increased levels of NGK2a-CD94^+^ TILs correlate with poor survival in CRC patients [256]. Although metastatic microsatellite-stable CRC patients do not respond to therapies that involve PD-1/PD-L1 blockade [257], a first phase I clinical trial studying an antibody against PD-1 (durvalumab) in combination with an antibody targeting CD94/NGK2a (monalizumab) is ongoing.

Immunotherapy checkpoints have been suggested as a good strategy to impair cancer progression [258], and strategies targeting both the innate and the adaptive immune systems show promising results in CCR [259]. CCL2, which is highly expressed in PDAC, is a chemoattractant for T cells, monocytes, and natural killer cells. CCL2 binds to its receptor, C-C Chemokine Receptor (CCR) 2, which is expressed in monocytes and controls its differentiation into TAMs [260]. CCR2 inhibition in combination with FOLFIRINOX in PDAC has been tried in phase I clinical trial, and the results showed that it was safe and well tolerated [261].

Another mention worthy molecule is CD47, an integrin-associated transmembrane protein. This integrin is overexpressed in solid cancers (e.g. CRC) and is correlated to a poor clinical outcome [262]. Both TAMs and DCs can express the CD47 receptor, signal regulatory protein alpha (SIRP*α*). The binding of CD47 to SIRP*α* inhibits phagocytosis of cancer these cells, enabling the tumor to evade immune destruction by first responder cells, such as macrophages [263]. Thus, restoring phagocytosis activity by antigen-presenting cells can enhance antigen priming of T cells. A phase I clinical trial recently described the use of the monoclonal antibody against CD47 Hu5F9-G4 in CRC and PDAC [264]. A Phase II study to evaluate Hu5F9-G4 in combination with cetuximab is ongoing in CRC [265].

As previously mentioned, the M2 macrophages are frequently found in TMEN. Since the intratumoral presence of Macrophage Colony-Stimulating Factor Receptor (CSFR)1^+^ macrophages correlates with the clinical aggressiveness of pancreatic neuroendocrine tumors [266], targeting CSFR1 signaling in TAMs represents an attractive strategy to eliminate these cells and block M2 polarization. A clinical trial using a monoclonal antibody against CSFR1, AMG 820, showed safety and tolerability in patients with advanced solid tumors, including CRC and PDAC [234]. However, since the study did not present significant tumor responses, it was terminated before enrollment into the dose-expansion phase. Preclinical studies have also examined the effects of CSFR1 inhibitors in combination with T-cell target therapies to improve efficacy in PDAC [267]. In fact, a clinical trial using pembrolizumab in combination with AMG 820 is ongoing in PDAC and in CRC, with an estimated date of completion in 2020 (Table 3).

### 8.3. Angiogenesis

Approaches focused on anti-angiogenesis cancer therapies have been studied in several clinical trials. In GC the results of trials with anti-VEGF were disappointing on the first line treatments (either with bevacizumab or with ramucirumab) [236, 268]. Interestingly, the use of ramucirumab (anti-VEGF2) in association with paclitaxel or in monotherapy showed a significant improvement on the overall survival of gastro-esophageal adenocarcinoma patients and has been approved in this setting [268, 269].

In HCC, the use of tyrosine kinases with antiangiogenic effects were the basis of systemic treatment. Since the first approved drug, sorafenib, several clinical studies showed improvement in clinical outcomes with regorafenib, ramucirumab or cabozantinib, expanding the repertoire of drugs that can be used in this particular disease [220, 270–272].

In CRC, the use of bevacizumab in association FOLFIRI (Folinic Acid, 5-FU, Irinotecan) or FOLFOX (Folinic Acid, 5-FU, oxaliplatin) showed a significant increase in overall survival, being nowadays the standard of care for patients in the metastatic stage of this disease [273]. Nonetheless, the use of bevacizumab as part of the adjuvant chemotherapy treatment in CRC patients was detrimental for survival [274]. Other drugs that change the tumoral angiogenesis, such as the VEGF 1 and 2 inhibitors ziv-aflibercept and ramucirumab, have shown an improvement in overall survival in patients with CRC when in combination with chemotherapy in second line setting after failure of a previous line of chemotherapy [275, 276].

## 9. Conclusions and Perspectives

Tumor masses are not cancer cells-centered entities that drive malignant progression. Instead, tumor development depends on the complex and intricate tapestry of cell-cell interactions where nontransformed cells of the TMEN play key role in cancer biology. We here summarized how stromal cells can impact tumor growth and progression as well as resistance to antitumor treatment. In fact, we show that most of these cells are important oncogenic drivers, frequently associated with poor prognosis. Therefore, the development of new therapeutic approaches directed to components of the TMEN still has a great unexplored potential. The main challenge on TMEN-directed approaches resides on the complexity of the interactions within the microenvironment, where the same cell type can have opposite effects in tumor growth and progression depending on its cell-to-cell interaction. This is not surprising considering the pleotropic diversity of all the stromal cells described here. Therefore, ideal targeted therapy is unlikely to be solely affecting a single cell type. Instead, the best therapeutic approaches should be those that are capable of tipping the whole balance in favor of tumor inhibition.

## Figures and Tables

**Table 1:** Clinical significance of stromal cells in GI cancers.

Stromal cell	Type of cancer	Levels	Clinical outcome	References
CAFs	GC	High	Metastasis	[154, 155]
PD-L1	PDAC	Expression	Poor prognosis	[156]
PD-L1	GC	Expression	Poor prognosis	[157–159]
PD-L1 and CD8^+^ T cells	GC/gastroesophageal	High	Lower survival	[160]
PD-L1 and PDL2	Esophageal	Expression	Poor prognosis	[122, 161]
M2 macrophages	PDAC	High	Lymphatic metastasis/poor prognosis	[162]
CD204^+^ (M2) macrophages	Esophageal	High	Poor DFS	[163]
M2 macrophage and %TRegs	PDAC	High	Lower survival	[155]
CAFs and M2 macrophages	CRC	Expression	Reduced survival	[148]
MDSC	PDAC	High	Progression	[164]
MDSC	GC/PDAC/esophageal	Low	Survival	[134]
Th2	PDAC	Presence	Reduced survival	[149]
TRegs	PDAC	High	Progression/poor prognosis	[165–168]
TCD3^low^/TReg^high^	CRC	Low/high	Lower survival	[169]
TRegs	CRC	High	Good prognosis	[170, 171, 172]
TRegs	HCC	High	Progression	[140, 173]
TRegs^low^/CD8^+^ TIL^high^	HCC	Low/high	High DFS	[140, 173]
TRegs^high^/CD8^+^ TIL^low^	GC	Ratio high	Lower survival	[174]
TRegs	GC and esophageal	High	Poor survival	[175]
DC and circulating myeloid DC	PDAC	High	Survival	[176]
CD4^+^/CD8^+^ TILs	PDAC	High	Good prognosis after surgery	[177]
CD4^+^/CD8^+^ TILs	Esophageal	High	Good prognosis	[178]
CD8^+^/CD45RO^+^ TILs	CRC	High	Good prognosis	[178, 179]
CD4^+^/CD8^+^/CD45RO^+^ TILs	GC	Low	Lymph node metastasis/lower survival	[180]
CD8^+^ and FoxP3^+^ TILs	GC (microsatellite unstable)	High	Good prognosis	[181]
M2 macrophages + CD8^+^ and FoxP3^+^ TILs	GC (microsatellite unstable)	High	Survival	[182]
M2 macrophages	GC	High	Poor survival and tumor progression	[145, 150]
NK^+^ cells	GC	High	Good prognosis	[183]

DFS: disease-free survival.

**Table 2:** Resistance to therapies targeting stromal components.

Drugs	Tumor type	Stromal-derived mediator
Doxorubicin and gemcitabine	PDAC	Hyaluronan [199]
Gemcitabine	PDAC	Sonic hedgehog [200]
Gemcitabine	PDAC	Alpha-SMA [193]
Gemcitabine	PDAC	HIF-alpha [208]
Gemcitabine	PDAC	CXCR4 [209]
Gemcitabine	PDAC	Snail [210]
Oxaliplatin	CRC	LncRNA19 [211]
5-FU	CRC	miR145, miR34-a [212]
Bevacizumab	CRC	VEGF [213]
Cisplatin	ESC	IL6R [214]
5-FU and oxaliplatin	GC	AKT, p38, and survivin [215]
Cisplatin	GC	miR-21 [216]

**Table 3:** Drugs targeting stroma components in clinical trials.

Drug and association	Tumor type	Molecular and cellular target	Mechanism	Study phase	ClinicalTrial.gov identifier
Sibrotuzumab	CRC, PDAC	FAP (CAF)	Desmoplasia [224]	II	NCT02198274
Simtuzumab + FOLFIRI	CRC, PDAC	LOXL2	Desmoplasia [229]	II	NCT01479465
Simtuzumab + gemcitabine	CRC, PDAC	LOXL2	Desmoplasia [229]	II	NCT01472198
Losartan + F-NOX	PDAC	TGF-beta1	Fibrosis [230]	II	NCT03563248 ^*∗*^
IPI-926 + gemcitabine	PDAC	SMO	Hedgehog pathway inhibition	II	NCT01130142
PEGPH20 + gemcitabine + nab-paclitaxel	PDAC	Hyaluronan	Desmoplasia [232, 233]	III	NCT02715804 ^*∗*^
PEGPH20 + FOLFIRINOX	PDAC	Hyaluronan	Desmoplasia [232, 233]	I	NCT01959139 ^*∗*^
Pembrolizumab + AMG820	CRC, PDAC	PD-1 (T cells) CSF1R (macrophage)	T-cell apoptosis	II	NCT02713529 ^*∗*^
Durvalumab + monalizumab	CRC	PD-1 (T cells) CD94/NGK2a	T-cell apoptosis	I	NCT02671435 ^*∗*^
AMG820	CRC, PDAC	CSF1R (macrophage)	M2 polarization [234]	I	NCT01444404
5F9 + cetuximab	CRC	CD47 (macrophage)	Restores macrophage phagocytosis	II	NCT02953782 ^*∗*^
Pembrolizumab + cisplatin + 5-fluorouracil	GC, GEJ	PD-1 (T cells)	T-cell apoptosis	III	NCT02494583 ^*∗*^
Pembrolizumab + paclitaxel	GC, GEJ	PD-1 (T cells)	T-cell apoptosis	III	NCT02370498 ^*∗*^
Ruxolitinib + capecitabine	PDAC	Janus 1 and Janus 2 (pancreatic stellate cells)	JAK-STAT3 pathway inhibition [235]	III	NCT02117479 ^#^ NCT02119663 ^#^
Nivolumab + ipilimumab	Upper GI	PD-1 (T cells) CTLA-4 (T cells)	Block T-cell inhibitory signals and activation of T cells	II	NCT02923934 ^*∗*^
Bevacizumab + cisplatin	GC	VEGF-A (endothelial cells)	Angiogenesis [236]	III	NCT00548548
Ramucirumab	Upper GI	Inhibits receptor tyrosine kinase (endothelial cells)	Angiogenesis	II	NCT02241720

GEJ: gastroesophageal junction. ^*∗*^ongoing; ^#^terminated.

## Abbreviations

GI: Gastrointestinal
PDAC: Pancreatic ductal adenocarcinoma
BM: Bone marrow
TMEN: Tumor microenvironment
BMDC: Bone marrow-derived cells
GC: Gastric cancer
CRC: Colorectal carcinoma
MMP: Metalloproteinase
MSC: Mesenchymal stem cells
VEGF: Vascular endothelial growth factor
IFN-*γ*: Interferon gamma
TNF-*α*: Tumor necrosis factor alpha
HIF-1*α*: Hypoxia-induced factor 1 alpha
S100A8/9: S100 calcium binding protein A8/A9
HCC: Hepatocellular carcinoma
PTEN: Phosphatase and tensin homolog
HSC: Hematopoietic stem cells
CAF: Cancer-associated fibroblasts
ECM: Extracellular matrix
TGF-*β*: Transforming growth factor beta
HGF: Hepatocyte growth factor
FSP1: Fibroblast-specific protein 1
Lkb1: Liver kinase B1
IL: Interleukin
JAK: Janus kinase
STAT3: Signal transducer and activator of transcription 3
PGE2: Prostaglandin E2
EMT: Epithelial-to-mesenchymal transition
PDGF: Platelet-derived growth factor
FAP: Fibroblast activation protein
CCL: C-C motif chemokine
CXCL: C-X-C motif ligand
CXCR: C-X-C motif receptor
GM-CSF: Granulocyte-macrophage colony-stimulating factor
SDF-1: Stromal cell-derived factor 1
*α*-SMA: Alpha smooth muscle actin
HUVEC: Human umbilical vein endothelial cells
ECs: Endothelial cells
AKT: Serine/threonine-protein kinase
ESC: Esophageal squamous carcinoma
LEC: Lymphatic endothelial cells
TIMP: Tissue inhibitor of metalloproteinases
PD-L1: Programmed death-ligand 1
IDO: Indoleamine 2,3-dioxygenase
NK: Natural killer
TRegs: T regulatory cells
FOXP3: Forkhead box P3
DC: Dendritic cells
Th2: Type II helper T cells
MDSC: Myeloid-derived suppressor cells
PD-1: Programmed death-1
Fas: Apoptosis-mediating surface antigen FAS
FasL: Fas ligand
MIF: Migration inhibitory factor
TIL: Tumor lymphocyte infiltrates
HA: Hyaluronic acid
Shh: Sonic hedgehog
5-FU: 5-Fluorouracil
TAM: Tumor-associated macrophages
LOXL2: Lysyl oxidase-like 2
SMO: Smoothened
FOLFIRINOX: Folinic acid, 5-FU, irinotecan, oxaliplatin
CD94/NGK2a: Natural killer cells antigen 94
CCR: C-C chemokine receptor
CSFR: Macrophage colony-stimulating factor receptor.
